# Metabolism-Associated Gene Signatures for FDG Avidity on PET/CT and Prognostic Validation in Hepatocellular Carcinoma

**DOI:** 10.3389/fonc.2022.845900

**Published:** 2022-01-31

**Authors:** Hyunjong Lee, Joon Young Choi, Je-Gun Joung, Jae-Won Joh, Jong Man Kim, Seung Hyup Hyun

**Affiliations:** ^1^ Department of Nuclear Medicine, Samsung Medical Center, Sungkyunkwan University School of Medicine, Seoul, South Korea; ^2^ Samsung Genome Institute, Samsung Medical Center, Sungkyunkwan University School of Medicine, Seoul, South Korea; ^3^ Department of Biomedical Science, College of Life Science, CHA University, Seongnam, South Korea; ^4^ Department of Surgery, Samsung Medical Center, Sungkyunkwan University School of Medicine, Seoul, South Korea

**Keywords:** hepatocellular carcinoma, prognosis, FDG PET/CT, lipid metabolism, glucose metabolism, gene signatures

## Abstract

**Introduction:**

The prognostic value of F-18 fluorodeoxyglucose positron emission tomography/computed tomography (FDG PET/CT) in hepatocellular carcinoma (HCC) was established in previous reports. However, there is no evidence suggesting the prognostic value of transcriptomes associated with tumor FDG uptake in HCC. It was aimed to elucidate metabolic genes and functions associated with FDG uptake, followed by assessment of those prognostic value.

**Methods:**

Sixty HCC patients with Edmondson–Steiner grade II were included. FDG PET/CT scans were performed before any treatment. RNA sequencing data were obtained from tumor and normal liver tissue. Associations between each metabolism-associated gene and tumor FDG uptake were investigated by Pearson correlation analyses. A novel score between glucose and lipid metabolism-associated gene expression was calculated. In The Cancer Genome Atlas Liver Hepatocellular Carcinoma dataset, the prognostic power of selected metabolism-associated genes and a novel score was evaluated for external validation.

**Results:**

Nine genes related to glycolysis and the *HIF-1* signaling pathway showed positive correlations with tumor FDG uptake; 21 genes related to fatty acid metabolism and the *PPAR* signaling pathway demonstrated negative correlations. Seven potential biomarker genes, *PFKFB4*, *ALDOA*, *EGLN3*, *EHHADH*, *GAPDH*, *HMGCS2*, and *ENO2* were identified. A metabolic gene expression balance score according to the dominance between glucose and lipid metabolism demonstrated good prognostic value in HCC.

**Conclusions:**

The transcriptomic evidence of this study strongly supports the prognostic power of FDG PET/CT and indicates the potential usefulness of FDG PET/CT imaging biomarkers to select appropriate patients for metabolism-targeted therapy in HCC.

## Introduction

Hepatocellular carcinoma (HCC) is the most representative malignancy in the liver and the fourth leading cause of cancer death worldwide (1). Hepatectomy, liver transplantation, and trans-arterial chemoembolization are conventional treatment options for HCC (2). Recently, targeted agents such as sorafenib and nivolumab are used in patients with advanced HCC as palliative treatment (3, 4). There have been many previous studies to explore predictive factors for the prognosis and treatment response of HCC. The concentration of alpha-fetoprotein, prothrombin induced by vitamin K absence or antagonist II, and histological grade are the most well-known prognostic factors (5–7).

F-18 fluorodeoxyglucose positron emission tomography/computed tomography (FDG PET/CT) is a robust imaging modality used to diagnose malignancy (8). CT or magnetic resonance imaging (MRI) is especially useful in the diagnosis of HCC due to its specific finding of HCC, early enhancement in arterial phase, and delayed washout in portal phase (9). The diagnostic performance of FDG PET/CT is inferior to CT and MRI as there are tumors with low FDG uptake or isometabolic uptake, which are difficult to discriminate from normal liver tissue (10, 11). Nevertheless, the prognostic value of FDG PET/CT in HCC has been revealed to be highly significant in many previous studies (12–15).

Prior research has suggested possible key proteins that affect tumor FDG uptake in HCC. Lee et al. showed that hexokinase II (*HK2*) is expressed in HCC in contrast to glucose transporter 1 (*GLUT1*), which is expressed in cholangiocarcinoma (16). They also reported genes related to cell survival to be associated with high FDG uptake (17). Recently, Xia et al. found that hypoxia-induced glucose transporters may contribute to FDG uptake in HCC based on radiogenomics results (18). However, previous studies recruited less than 20 patients. In addition, there are no previous reports suggesting the prognostic value of transcriptomes associated with FDG uptake in HCC tumors. It is expected that exploring metabolic genes or functions associated with FDG uptake and evaluating their prognostic value can strongly support not only the prognostic value of FDG PET/CT but also reveal molecular functions affecting FDG uptake in HCC.

In this study, we aimed to elucidate significant metabolic genes and functions associated with FDG uptake in HCC transcriptomes as an RNA-sequencing dataset. Subsequently, the prognostic value of the genes and gene set expression scores were assessed. Ultimately, transcriptomic evidence highlighted the prognostic power of FDG PET/CT in HCC.

## Methods

### Subjects

Between May 2009 and August 2015, patients who underwent curative surgery for HCC and pretreatment FDG PET/CT were enrolled. We identified 120 eligible samples (60 tumor tissues and 60 paired normal liver tissues) in 60 patients (49 males and 11 females; mean age, 58.1 ± 8.8 years) from the Samsung Medical Center Biobank. A single nodule of HCC was present in all patients. Samples were obtained after surgical resection prior to radiation or chemotherapy and were stored in liquid nitrogen. In all tumor samples, the pathological diagnosis and Edmondson–Steiner grade were verified by a pathologist. Only tumor samples with Edmondson–Steiner grade II were included in the study cohort, and the other samples of other grades were excluded, as there is a high variation of FDG avidity depending on the cell differentiation grade. Samples were collected in accordance with the guidelines issued by the ethics committee of our institution, and written informed consent was obtained from all patients. Our institutional review board approved this retrospective study (IRB #2017-04-022). Demographic and clinical characteristics and survival data were obtained from electronic medical records.

### FDG PET/CT Acquisition and Image Analysis

All patients fasted for at least 6 hours and had blood glucose levels of less than 200 mg/dL at the time of FDG PET/CT. Whole-body PET and CT images from the basal skull to mid-thigh were acquired 60 minutes after 5.0 MBq/kg FDG injection without intravenous or oral contrast on a dedicated PET/CT scanner (Discovery STE, GE Healthcare, Milwaukee, WI). Continuous spiral CT was performed with a 16-slice helical CT (140 keV, 30–170 mA). An emission scan was then obtained from head to thigh for 2.5 minutes per frame in 3-dimensional mode. PET images were reconstructed using CT, and attenuation correction was performed using the ordered-subsets expectation-maximization algorithm with 20 subsets and 2 iterations (matrix size, 128×128; voxel size, 3.9×3.9×3.3 mm).

All images were reviewed by a board-certified nuclear medicine physician using volume viewer software on a GE Advantage Workstation, version 4.7. The maximum standardized uptake value (SUVmax) of the primary tumor was measured using a spherical volume of interest over the primary tumor. The mean SUV (SUVmean) of the normal liver was obtained by taking the average of the three 2-cm-diameter spherical VOIs (two in the right lobe and one in the left lobe). Tumor FDG avidity was measured by tumor-to-normal liver SUV ratio (TLR), calculated with the following equation: TLR = SUVmax of the tumor/SUVmean of the normal liver.

### RNA Sequencing

Frozen sections from each tissue sample were homogenized in TRIZOL reagent (Invitrogen, Carlsbad, CA, USA). Total RNA was extracted using a standard chloroform protocol followed by purification with the Qiagen RNeasy Mini Kit (QIAGEN Inc, Valencia, CA. USA). RNA integrity was evaluated using RNA 6000 Nano LabChips on an Agilent 2100 Bioanalyzer (Agilent Technologies, Foster City, CA, USA). RNA purity was assessed by the ratio of spectrophotometric absorbance at 260 and 280 nm (A260/280 nm) using NanoDrop ND-1000 (NanoDrop Inc, Wilmington, DE, USA). Library construction for RNA sequencing was performed using a Truseq RNA Sample Preparation v2 Kit (Illumina). Isolated total RNA was used in a reverse transcription reaction with poly (dT) primers using SuperScriptTM II Reverse Transcriptase (Invitrogen) according to the manufacturer’s protocol. Briefly, an RNA sequencing library was prepared by cDNA amplification, end-repair, 3’ end adenylation, and adapter ligation. Library quality and quantity were measured using the Agilent 2100 Bioanalyzer and Qubit. Sequencing of the RNA library was carried out using the 100 bp paired-end mode of the TruSeq Rapid PE Cluster Kit and the TruSeq Rapid SBS Kit (Illumina). Reads from the FASTQ files were mapped to the hg19 human reference genome using TopHat version 2.0.6 (http://tophat.cbcb.umd.edu/). Raw read counts mapped to genes were measured using the BAM format file by HTSeq, version 0.6.1 (https://htseq.readthedocs.io). Read counts were normalized using the TMM (Trimmed Mean of M-values normalization) method. The expression of genes in tumor tissue was divided by that in normal liver tissue to calculate the fold change of gene expression. The fold changes were normalized by log_2_ transformation.

### Gene Sets and Molecular Functions Associated With FDG Uptake in HCC

Metabolism-associated genes encoding proteins involved in glucose and lipid metabolism were selected from the Molecular Signature Database (mSigDB). A set of 404 genes was manually curated for further transcriptomic analysis. Pearson correlation analyses were performed between the expression of metabolism-associated genes and TLRs. All genes with positive or negative correlations (*p* < 0.05) were defined as metabolism-associated genes related to tumor FDG uptake. In each gene set, REACTOME and KEGG enrichment analysis was conducted to investigate the molecular pathways associated with tumor FDG uptake using the “signatureSearch” package in R/Bioconductor (19). In each metabolism-associated gene set, a gene expression signature score (GESS) was defined as the mean z-score of each gene expression. We calculated a novel balance score between glucose and lipid metabolism-associated gene expression in HCC (metabolic balance score) by subtracting the GESS of lipid metabolism from the GESS of glucose metabolism. The concept of subtracting the average of z-score of each gene set was already applied in a previous study to evaluate a value of epithelial-mesenchymal transition score in lung cancer (20).

### Prognostic Validation of Metabolism-Associated Genes

Among the investigated metabolism-associated genes, those with significant correlation with TLR were selected as subject genes for further analysis. Prognostic validation of metabolism-associated genes was performed in The Cancer Genome Atlas Liver Hepatocellular Carcinoma (TCGA-LIHC) dataset. Clinical and gene expression data of TCGA-LIHC patients were obtained from cBioPortal using “cgdsr” and “TCGAbiolinks” packages in R/Bioconductor. Among the whole dataset, 325 patients with disease-free survival (DFS) data and 376 patients with overall survival (OS) data were included in this study. As previously described, the GESS of glucose metabolism, the GESS of lipid metabolism, and the metabolic balance score between them were calculated. A zero for metabolic balance score was used as a cutoff to classify subjects into two risk stratification groups.

Regarding multivariable Cox regression analysis, variables with a *p*-value less than 0.1 in univariable analyses were included. Variables with collinearity were excluded. Metabolism-associated genes with univariable *p*-values less than 0.1 for both DFS and OS were selected as potential components of the biomarker gene set. The prognostic index (PI) was developed using Cox proportional hazards regression model to validate risk stratification with the biomarker gene set. Risk stratification groups were divided by a median value of PI. The expression of each potential biomarker gene was compared between each group using independent t-test. Gene mutation data were downloaded from the genomic data commons (https://gdc.cancer.gov/), and a mutation annotation format file was constructed using the “read.maf” function included in the “maftools” package in R/Bioconductor (21). The difference of *TP53* and *CTNNB1* mutations between risk groups was evaluated by Chi-square test. All the statistical analyses were performed using R software (v4.0.4, R Foundation for Statistical Computing, Vienna, Austria). A *p*-value less than 0.05 was considered statistically significant.

## Results

### Patients

The patient characteristics are summarized in **Table 1**. In 31 patients (51.7%), tumors with TLRs greater than or equal to median value of TLR presented the high TLR phenotype. In the remaining 29 patients (48.3%), tumors with TLRs less than median value of TLR were assigned to the low TLR phenotype. The median value of TLR was 1.7. TLRs ranged from 1.7 to 6.8 (mean: 2.8) for high TLR tumors and from 1.1 to 1.7 (mean: 1.4) for low TLR tumors. Milan criteria compliance (*p* = 0.014), young age (*p* = 0.025), large tumor size (*p* = 0.005), and the presence of microvascular invasion (*p* < 0.001) were significantly associated with high TLR phenotype. There were no significant associations between TLR and gender, normal liver SUV, HCC etiology, or presence of liver cirrhosis.

**Table 1:** Clinical characteristics of patients.

Characteristics	Overall	Low TLR	High TLR	*p*
	(n = 60)	(n = 29)	(n = 31)	
Age (range, years)	58.1 (42-76)	60.7 (43-76)	55.6 (42-73)	0.025
Sex, male	49 (81.7%)	24 (82.8%)	25 (80.6%)	1.000
Tumor size (cm)	4.4 ± 1.4	3.8 ± 1.3	4.9 ± 1.3	0.005
Milan criteria compliance	40 (66.7%)	24 (82.8%)	16 (51.6%)	0.014
Tumor SUV	4.5 ± 2.6	3.1 ± 0.5	5.9 ± 3.1	<0.001
Normal liver SUV	2.2 ± 0.3	2.2 ± 0.3	2.1 ± 0.3	0.166
TLR	2.1 ± 1.2	1.4 ± 0.2	2.8 ± 1.4	<0.001
Etiology				
HBV	46 (76.7%)	23 (79.3%)	23 (74.2%)	0.431
HCV	5 (8.3%)	1 (3.4%)	4 (12.9%)	
Alcohol and others	9 (15.0%)	5 (17.2%)	4 (12.9%)	
Cirrhosis				
No	38 (63.3%)	20 (69.0%)	18 (58.1%)	0.544
Yes	22 (36.7%)	9 (31.0%)	13 (31.9%)	
Microvascular invasion				
No	30 (50.0%)	22 (75.8%)	8 (25.8%)	<0.001
Yes	30 (50.0%)	7 (24.2%)	23 (74.2%)	

Data are numbers of patients (proportion) or mean values ± standard deviation. Tumor with TLR more than median value was assigned high TLR phenotype.

SUV, standardized uptake value; TLR, Tumor-to-normal liver SUV ratio.

### Metabolism-Associated Genes and Molecular Functions Related to Tumor FDG Uptake

**Figure 1:**
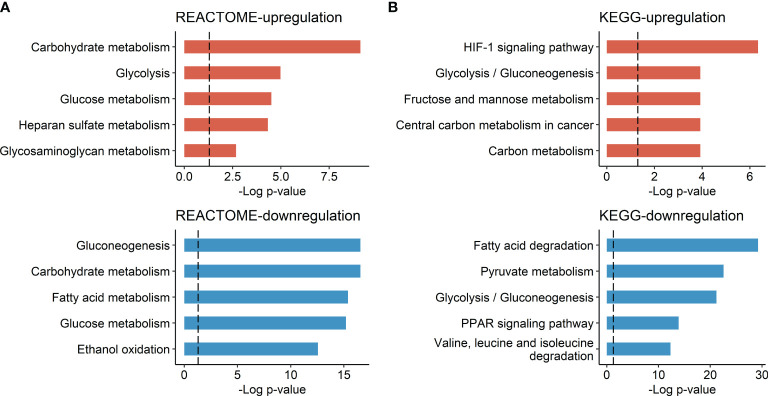
Molecular pathways associated with HCC tumor FDG uptake. **(A)** Molecular pathways listed in the REACTOME database and **(B)** molecular pathways listed in the KEGG database. Bar plots represent the negative log *p*-value of each molecular pathway.

There were 42 genes with significant positive correlations and 87 genes with significant negative correlations (*p* < 0.05) with tumor FDG uptake. The list of genes is described in **Supplementary Table 1**. Gene set enrichment analysis according to TLR using the REACTOME and KEGG databases demonstrated upregulated glucose metabolism, including glycolysis and hypoxia-inducible factor-1 (*HIF-1*) signaling pathway upregulation, and downregulated lipid metabolism, including fatty acid metabolism and peroxisome proliferator-activated receptor (*PPAR*) signaling pathway downregulation, in HCC with high TLR phenotype (**Figure 1**).

Considering the overlap of genes in these molecular pathways, the upregulated gene sets involved in glycolysis and *HIF-1* signaling were merged into a glucose metabolism-associated gene set. The downregulated gene sets involved in fatty acid metabolism and the *PPAR* signaling pathway were merged into a lipid metabolism-associated gene set. There were nine genes with positive correlation among the glucose metabolism-associated gene set and 21 genes with negative correlation among the lipid metabolism-associated gene set. The list of glucose metabolism-associated genes and lipid metabolism-associated genes related to tumor FDG uptake are described in **Supplementary Tables 2, 3**, respectively.

**Figure 2:**
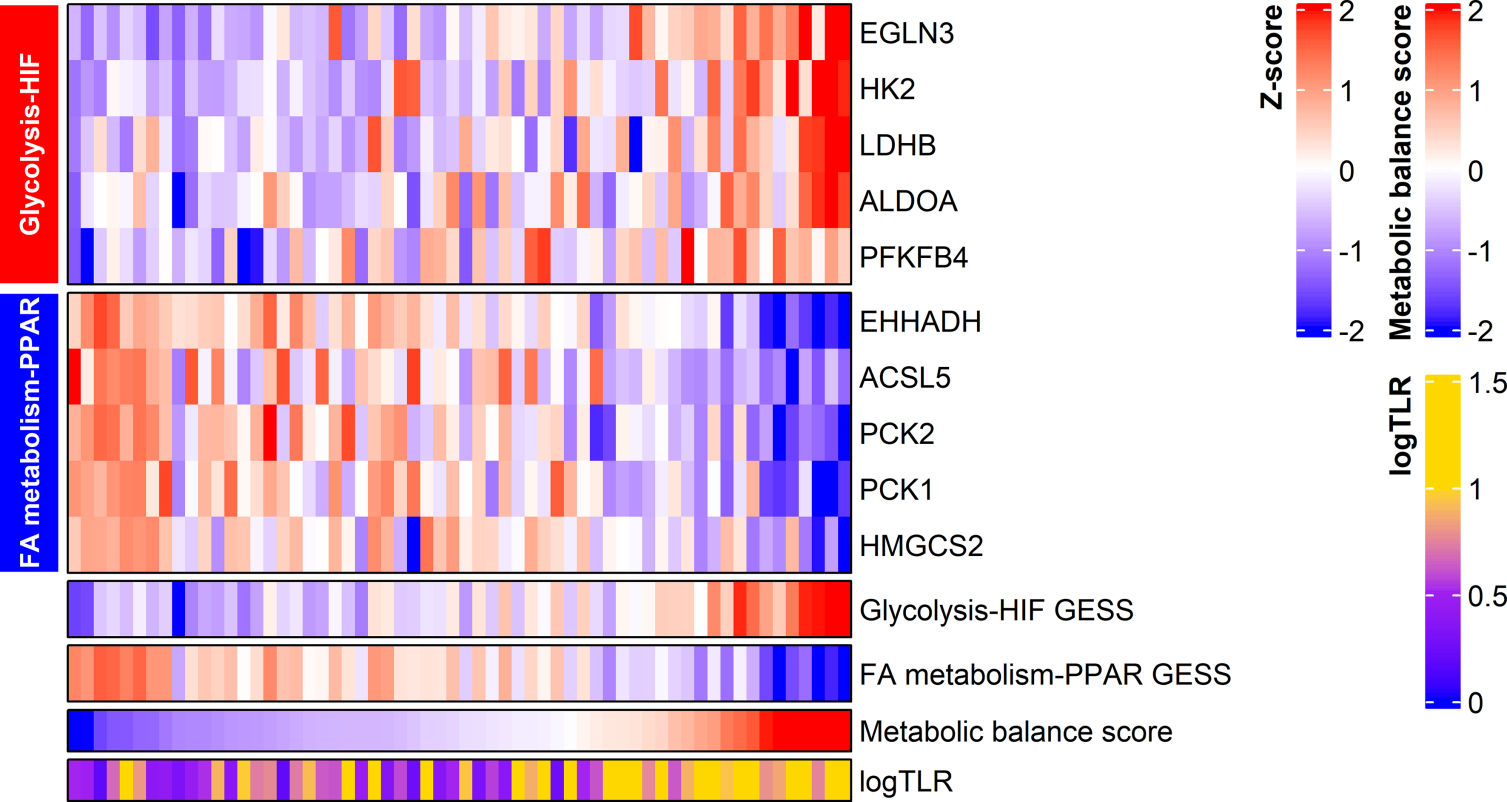
Expression of metabolism-associated genes related to HCC tumor FDG uptake. The expression of the top five genes from each gene set are displayed in the heatmap. Metabolic balance score showed a positive correlation with TLR in HCC. logTLR, tumor-to-normal liver standardized uptake value ratio in log scale base 2; HIF, hypoxia-inducible factor-1; FA, fatty acid.

The GESS of glucose metabolism showed a positive correlation with tumor FDG uptake (r = 0.607 and *p* < 0.001). The GESS of lipid metabolism showed a negative correlation with tumor FDG uptake (r = -0.562 and *p* < 0.001). The metabolic balance score showed a positive correlation with tumor FDG uptake (r = 0.639, *p* < 0.001). A heatmap visualized the expression of the top 5 genes with high correlation coefficients, the GESSs of each metabolism, and TLR values according to the metabolic balance score (**Figure 2**).

### Prognostic Validation of Metabolism-Associated Genes in the TCGA-LIHC Dataset

By means of univariable Cox regression analysis for DFS, *PFKFB4, ALDOA, EGLN3, CYP4A22, PCK1, ACADL, CYP4A11, EHHADH, GAPDH, HMGCS2*, and *ENO2* genes were found to have *p*-values of less than 0.1 in Wald test. In multivariate Cox regression analysis for DFS, *PFKFB4* was an independent prognostic gene (**Table 2**). In univariable Cox regression analysis for OS, *PFKFB4, EGLN3, ALDOA, GAPDH, HK2, ENO2, PFKFB3, HIF1A, HMGCS2, EHHADH, ECI1*, and *LDHB* genes had *p*-values less than 0.1. In multivariable Cox regression analysis for OS, *PFKFB4, EGLN3, GAPDH, HMGCS2*, and *ECI1* were independent prognostic genes (**Table 3**).

**Table 2:** Cox regression analysis of disease-free survival for metabolism-associated genes.

Genes	Disease-free survival					
	Univariate			Multivariate		
	HR	95% CI	*P*	HR	95% CI	*P*
PFKFB4	1.192	1.081-1.314	< 0.001	1.208	1.049-1.390	0.009
ALDOA	1.203	1.060-1.366	0.004	1.159	0.905-1.484	0.243
EGLN3	1.094	1.028-1.166	0.005	1.061	0.967-1.164	0.212
CYP4A22	0.939	0.892-0.988	0.016	0.962	0.864-1.071	0.476
PCK1	0.942	0.897-0.990	0.019	0.992	0.922-1.068	0.832
ACADL	0.948	0.904-0.994	0.026	0.951	0.982-1.014	0.123
CYP4A11	0.937	0.883-0.995	0.033	1.061	0.941-1.196	0.335
EHHADH	0.905	0.825-0.992	0.033	1.033	0.888-1.202	0.673
GAPDH	1.185	1.005-1.397	0.043	0.889	0.681-1.161	0.388
HMGCS2	0.922	0.851-0.999	0.046	0.971	0.851-1.107	0.655
ENO2	1.085	0.998-1.179	0.055	0.884	0.769-1.015	0.081

HR, hazard ratio; CI, confidence interval.

**Table 3:** Cox regression analysis of overall survival for metabolism-associated genes.

Genes	Overall survival					
	Univariate			Multivariate		
	HR	95% CI	*P*	HR	95% CI	*P*
PFKFB4	1.315	1.181-1.465	< 0.001	1.174	1.004-1.373	0.045
EGLN3	1.188	1.103-1.279	< 0.001	1.134	1.006-1.278	0.039
ALDOA	1.329	1.162-1.520	< 0.001	Excluded due to collinearity		
GAPDH	1.445	1.202-1.738	< 0.001	1.383	1.058-1.809	0.018
HK2	1.147	1.061-1.241	< 0.001	1.104	0.907-1.134	0.804
ENO2	1.157	1.060-1.263	0.001	0.833	0.705-0.984	0.031
PFKFB3	1.168	1.062-1.286	0.001	1.115	0.954-1.304	0.172
HIF1A	1.228	1.070-1.411	0.004	0.937	0.754-1.165	0.560
HMGCS2	0.907	0.844-0.975	0.008	0.890	0.795-0.997	0.043
EHHADH	0.908	0.819-1.006	0.065	1.124	0.950-1.328	0.173
ECI1	0.804	0.628-1.028	0.082	0.727	0.538-0.984	0.039
LDHB	1.117	0.983-1.271	0.090	0.989	0.850-1.152	0.891

HR, hazard ratio; CI, confidence interval.

**Figure 3:**
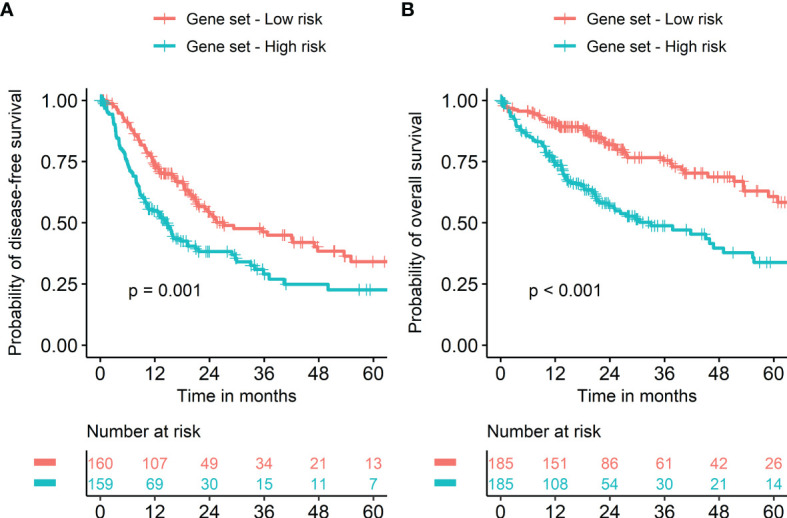
Survival of HCC patients according to the biomarker gene expression signature. Risk groups were classified according to prognostic index with seven biomarker genes (*PFKFB4, ALDOA, EGLN3, EHHADH, GAPDH, HMGCS2*, and *ENO2*). Kaplan‐Meier curves showed a significantly worse prognosis in patients with high-risk gene expression signatures compared to those with low-risk gene expression signatures in terms of both disease-free **(A)** and overall survival **(B)**.

**Figure 4:**
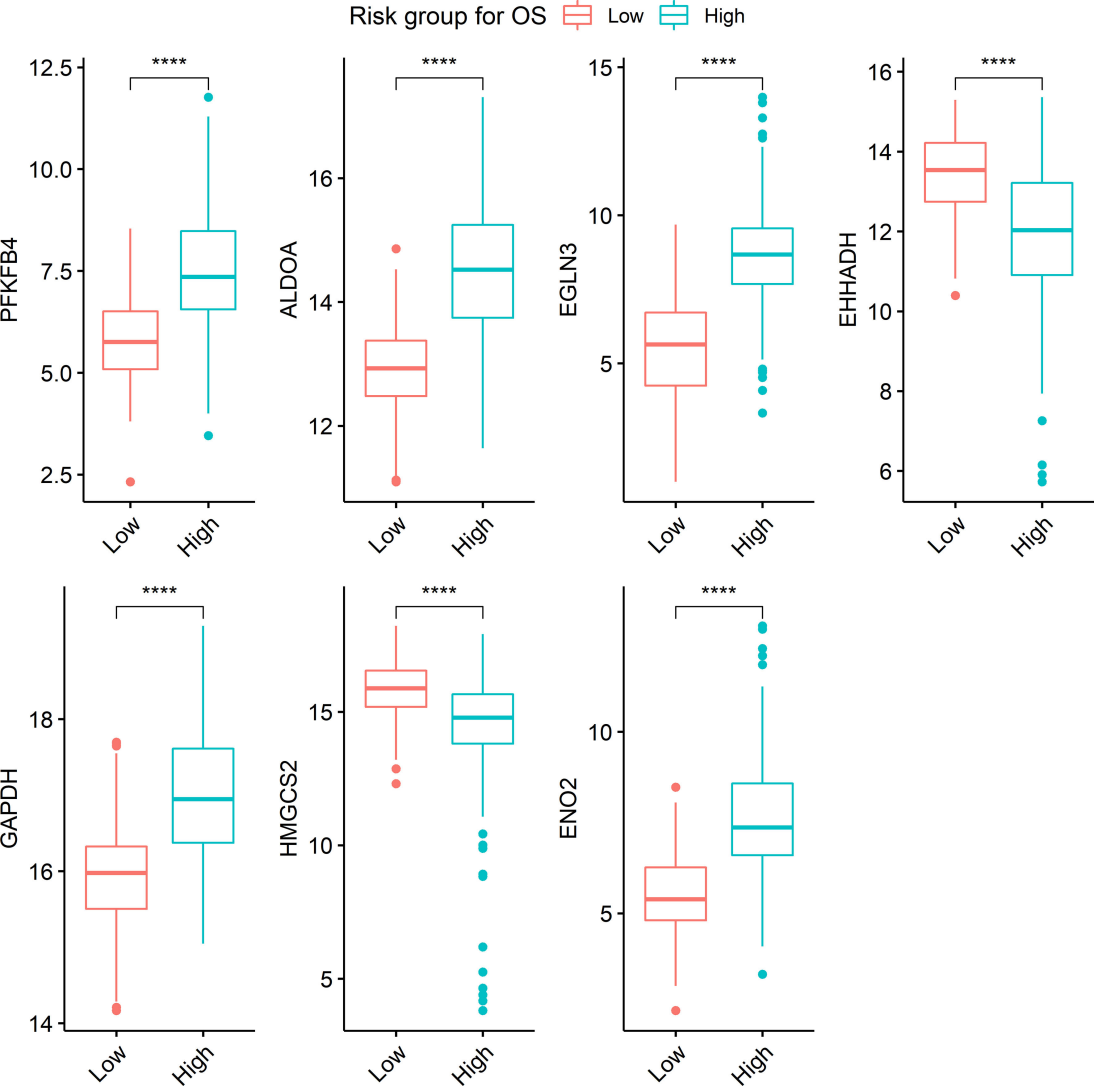
Boxplots visualizing the expression level of genes according to risk groups. ****The expression level of each gene was significantly different between risk groups (*p* < 0.001). Boxplots represent the expression level of seven genes, *PFKFB4, ALDOA, EGLN3, EHHADH, GAPDH, HMGCS2*, and *ENO2*.

Seven metabolism-associated genes, including *PFKFB4, ALDOA, EGLN3, EHHADH, GAPDH, HMGCS2*, and *ENO2*, were potential prognostic biomarkers for HCC. Kaplan-Meier curves showed a significantly worse DFS (*p* = 0.001) and OS (*p* < 0.001) in patients with high-risk GESS compared to those with low-risk GESS (**Figure 3**). The expression levels of *PFKFB4, ALDOA, EGLN3, GAPDH*, and *ENO2* were significantly higher in the high-risk group for OS, while the expression levels of *EHHADH* and *HMGCS2* were significantly lower in the high-risk group for OS (*p* < 0.001, **Figure 4**).

**Figure 5:**
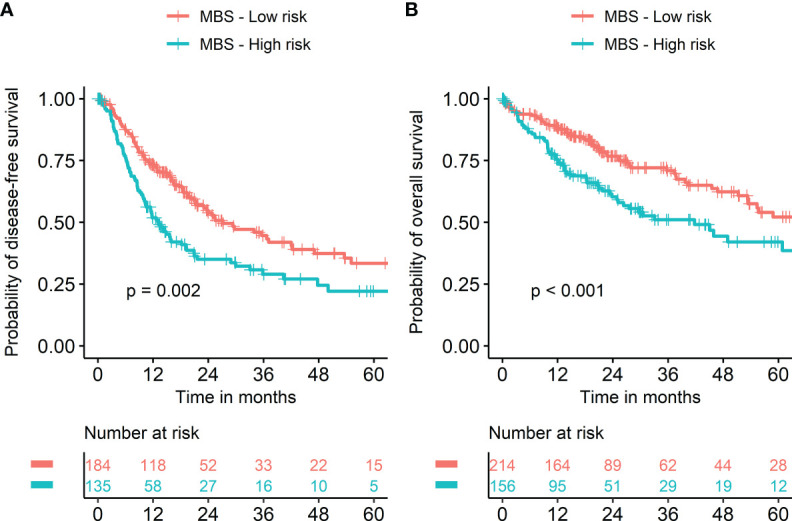
Kaplan‐Meier survival curves according to metabolic balance score. Risk groups were classified into two groups using metabolic balance score (MBS). Kaplan‐Meier curves showed significantly worse prognosis in patients with high MBS compared to those with low MBS in terms of both disease-free **(A)** and overall survival **(B)**.

Stratifying metabolic balance scores using a cutoff of zero demonstrated significant differences in DFS (*HR* = 1.22 and *p* = 0.002) and OS (*HR* = 1.33 and *p* < 0.001) according to metabolic dominance (glucose versus lipid, **Figure 5**). The proportion of high-risk patients with *TP53*-mutant HCC was significantly higher than that of those with wild-type *TP53* (57.9% vs. 36.7%, *p* < 0.001, **Supplementary Figure 1A**). The proportion of low-risk patients with *CTNNB1-*mutant HCC was significantly higher than that of patients with wild-type *CTNNB-1* (74.7% vs. 53.0%, *p* < 0.001, **Supplementary Figure 1B**). A heatmap was used to visualize the expression level of 30 metabolism-associated genes according to risk group (**Supplementary Figure 2**).

## Discussion

FDG PET/CT is the representative imaging modality to explore biologic tumor characteristics. Particularly in HCC, FDG PET/CT imaging findings have unique characteristics compared to other malignancies. Although many kinds of malignancies show high FDG uptake, it is not uncommon for HCC tumors to show low FDG uptake or isometabolic uptake, which is difficult to discriminate from normal liver tissue. This is not only due to the relatively high physiologic uptake of liver tissue but also due to the biologic characteristics of HCC. Therefore, previous studies have attempted to investigate proteins and genes affecting FDG uptake in HCC. Izuishi et al. showed that increased levels of *GLUT1* and decreased levels of glucose-6-phosphatase are associated with high HCC FDG uptake (22). Chen et al. revealed an inverse correlation between the expression of fructose 1,6-bisphosphatase 1 and FDG uptake (23). Lee et al. investigated the characteristics of gene expression profiles according to FDG uptake in ten patients with HCC (17). Contrary to the aforementioned research, the present study performed an explorative investigation of metabolism-associated gene expression within 60 patients, which is the largest cohort for a radiogenomics HCC study to the best of our knowledge.

In this study, molecular pathways of glycolysis and *HIF-1* signaling were revealed to be positively associated with HCC tumor FDG uptake. Glycolysis is the most representative mechanism to affect tumor glucose uptake. Tumor cells promote *HIF-1* signaling to resist hypoxic conditions. The *HIF-1* signaling pathway enhances the anaerobic glycolytic pathway to provide energy for tumor cells (24). The result of this study implies that resistance to hypoxic conditions mainly contributes to glucose uptake in HCC. Although there are previous reports that *HIF-1* signaling correlates with FDG uptake in other types of cancer, such as lung cancer and breast cancer (25, 26), there was no transcriptomic evidence of the association between *HIF-1* signaling and FDG uptake in HCC.

Notably, *PFKFB4* showed a significant, positive correlation with FDG uptake. It was a significant, independent prognostic gene for survival in the TCGA-LIHC dataset. *PFKFB* enzymes produce fructose-2,6-bisphosphate, which activates 6-phosphofructo-1-kinase, a rate-limiting enzyme in glycolysis (27). It was revealed as a poor prognostic factor in HCC (28). Interestingly, *EGLN3* was another significant, independent prognostic gene for OS in the TCGA-LIHC dataset among the other metabolism-associated genes related to tumor FDG uptake. *PHD3*, which is a protein encoded by the *EGLN3* gene, has tumor suppressor functions in various cancer types (29–31). However, there is controversy surrounding the prognostic value of *PHD3* in cancer. Previous studies suggested that *PHD3* downregulation is correlated with HCC aggressiveness and poor prognosis (32, 33). On the other hand, some reports indicated that increased *PHD3* had an association with poor prognosis in other cancer subtypes (34, 35). The results of this study support the effect of increased *PHD3* on unfavorable prognosis. *EGLN3*, a target gene of the *HIF-1* protein, induces positive feedback following enhanced *HIF-1* activity (36). In this regard, increased *PHD3* expression under hypoxia was shown to enhance cancer cell survival and the progression of disease (37). In addition, *PFKFB4* is induced by *HIF-1* activation in hypoxic conditions (38). In brief, it is suggested that upregulation of the glycolytic pathway *via PFKFB4* in hypoxic conditions mainly affects poor prognosis in HCC. Furthermore, the results of the present study strongly support previous knowledge that high tumor FDG uptake in HCC is associated with poor prognosis and hypoxic tumor microenvironmental conditions (14, 15).

Fatty acid metabolism and *PPAR* signaling were downregulated functions alongside increased FDG uptake. This result is readily understandable, as the *PPAR* signaling pathway regulates fatty acid oxidation (39). Tanaka et al. suggested the de-differentiation of HCC to be correlated with reduced fatty acid oxidation and increased glycolysis (40). In this aspect, the present study is consistent with the well-established knowledge that poorly differentiated HCCs demonstrate high FDG uptake (41). In addition, *HMGCS2* and *ECI1*, involved with fatty acid metabolism, are correlated with good prognosis with respect to OS, supported by previous knowledge that the suppression of fatty acid oxidation promotes the growth and metastasis of HCC (42). Furthermore, this result supports that PET/CT using C-11 acetate or F-18 fluorocholine tracers to visualize HCC with low FDG uptake due to enhanced fatty acid metabolism (43, 44).

We calculated the unified metabolic reprogramming scale representing the balance between glucose versus lipid metabolism gene expression in HCC. A high metabolic balance score was hypothesized to represent the metabolic shift to glycolytic process from fatty acid metabolism. This score showed an excellent correlation with tumor FDG uptake. The cutoff of zero, which is supposed as the balanced state of glucose versus lipid metabolism, showed good prognostic stratification. In brief, metabolic shift to glucose metabolism from lipid metabolism contributes to tumor FDG uptake and poor prognosis in HCC. Furthermore, there were significant differences in *TP53* and *CTNNB1* mutation between risk groups according to metabolic balance score. This suggests that metabolic characteristics may be associated with the genetic mutation profile, consistent with a previous report of HCC transcriptome classification demonstrating the associations between *TP53* mutation and the cell cycle as well as those between *CTNNB1* mutation and the *Wnt* pathway (45). In addition, there are similar studies reporting enhanced glycolysis in *TP53*-mutant HCCs and enhanced fatty acid oxidation in *CTNNB1*-mutant HCCs (46, 47).

We selected seven gene signatures associated with tumor FDG uptake in HCC. A prognostic model with those genes showed excellent stratification for both DFS and OS. In addition, those genes may have significance in selecting potential candidates for individualized therapy in terms of precision medicine. Sorafenib, as a kinase inhibitor, and nivolumab, as an immune checkpoint inhibitor, have been recently used for molecular targeted therapy (3, 4). The therapeutic effect of sorafenib is decreased under hypoxic conditions (48). In addition, suppressing glycolysis results in re-sensitizing HCC cells to sorafenib (49). As glycolytic activity and *HIF-1* signaling are activated in tumors with high FDG uptake, the application of sorafenib may be considered referring to FDG PET/CT findings. In particular, CD147 is a favorable therapeutic target with respect to the metabolic reprogramming of HCC. Within glucose metabolism, CD147 promotes tumor growth through the regulation of glycolysis *via* degradation of p53 protein (50). In addition, p53 downregulates the expression of *PFKFB4*, which showed an excellent correlation with tumor FDG uptake and good prognostic power in the present study (51). Within fatty acid metabolism, CD147 induces tumor growth by regulating fatty acid oxidation *via* inhibition of PPAR-alpha. In brief, inhibition of CD147 may be a novel therapeutic strategy for metabolism modulation (52). One clinical study showed the treatment effects of ^131^I-metuximab, which is a radioimmunoconjugate of iodine-131, and monoclonal antibody targeting CD147. It provided profit in survival rate and recurrence rate in HCC patients that underwent radiofrequency ablation (53). This study indicates that FDG PET/CT may be a good diagnostic modality to select candidates for metabolism-targeted therapy. CD147-targeted therapy to inhibit glycolysis and disinhibit fatty acid metabolism should be considered for patients with high tumor FDG uptake. Further study is warranted to evaluate the role of FDG PET/CT imaging biomarkers to select appropriate patients for metabolism-targeting therapy.

In brief, the present study has several clinical implications. First, conventional microscopic assessment of HCC is not fully standardized so that there is limitation to predict prognosis and select individualized therapeutic option accurately. The concrete transcriptomic evidence suggested in this study may be validated and utilized in personalized medicine in terms of tumor metabolism. Second, FDG PET/CT as a non-invasive functional imaging is commonly performed in initial workup for malignant disease including HCC. It is usually conducted prior to biopsy or surgery which allows obtaining histological samples. Metabolic characteristics of HCC as well as presence of metastasis provided by FDG PET/CT will contribute to predict prognosis and plan further treatment or follow-up. Furthermore, it is expected to support surrogate information for histopathologic findings as TLR showed a good association with microvascular invasion which is an important prognostic factor (data not shown). Third, transcriptomic examination is not commonly utilized in clinical field due to its high cost and requirement of high-end analytic instruments. FDG PET/CT may provide information of glucose metabolism which can be obtained from genomic analysis. In addition, a useful complementary information for lipid metabolism of HCC can be obtained with other tracers such as F-18 fluorocholine (54, 55).

There are several limitations to this study. First, a prognostic validation analysis could not be performed in subjects with both FDG PET/CT imaging and RNA-seq data due to a limited sample size. Further study as an internal and external validation is warranted to analyze prognostic power. Second, we have focused on metabolism-associated genes related to tumor FDG uptake. Although there are many biological functions and pathways that affect tumor FDG uptake in HCC, we did not cover the whole transcriptome. Nevertheless, the limited scope of subject genes aided in the analysis of significant molecular pathways by removing genes irrelevant to key metabolic processes.

In conclusion, metabolism-associated genes and molecular functions associated with tumor FDG uptake were explored in HCC. Increased tumor FDG uptake was found to be associated with glycolysis and *HIF-1* signaling pathway upregulation, whereas fatty acid metabolism and *PPAR* signaling were downregulated. Metabolic balance score representing the gene expression balance between glucose versus lipid metabolism in HCC showed a good association with tumor FDG uptake. It also showed excellent prognostic power in the TCGA-LIHC dataset. Seven genes, *PFKFB4, ALDOA, EGLN3, EHHADH, GAPDH, HMGCS2*, and *ENO2*, related to tumor FDG uptake were revealed to have a good prognostic value for survival in HCC. This study suggested key metabolic pathways to potentially affect tumor FDG uptake in HCC. The transcriptomic evidence of this study strongly supports the prognostic power of FDG PET/CT and indicates the potential usefulness of FDG PET/CT imaging to select appropriate HCC patients for metabolism-targeted therapy.

## Data Availability Statement

The datasets presented in this study can be found in online repositories. The names of the repository/repositories and accession number(s) can be found below: NCBI under BioProject PRJNA794275.

## Ethics Statement

The studies involving human participants were reviewed and approved by Samsung Medical Center Institutional Review Board. Written informed consent for participation was not required for this study in accordance with the national legislation and the institutional requirements.

## Author Contributions

HL, JYC, and SH designed the study. J-GJ, J-WJ, and JK contributed to data collection. HL and SH performed data analysis and interpretation. HL and SH drafted the article. JYC and J-GJ provided critical revision of the article. All authors contributed to the article and approved the submitted version.

## Funding

This research was supported by the Basic Science Research Program through the National Research Foundation of Korea (NRF) funded by the Ministry of Science and ICT (Grant No. NRF-2017R1A2B4006598).

## Conflict of Interest

The authors declare that the research was conducted in the absence of any commercial or financial relationships that could be construed as a potential conflict of interest.

## Publisher’s Note

All claims expressed in this article are solely those of the authors and do not necessarily represent those of their affiliated organizations, or those of the publisher, the editors and the reviewers. Any product that may be evaluated in this article, or claim that may be made by its manufacturer, is not guaranteed or endorsed by the publisher.

## Supplementary Material

Supplementary Figure 1 The proportion of high-risk patients according to *TP53* and *CTNNB1* mutation status. **(A)** The proportion of high-risk patients with *TP53*-mutant HCC was significantly higher than that of those with wild-type *TP53* (57.9% vs. 36.7%, *p* < 0.001). **(B)** The proportion of low-risk patients with *CTNNB1-*mutant HCC was significantly higher than that of patients with wild-type *CTNNB-1* (74.7% vs. 53.0%, *p* < 0.001). Mut (+), mutant; WT, wild type.

Supplementary Figure 2 The expression of 30 metabolism-associated genes according to prognostic index. Subjects were plotted in increasing order of prognostic index in the heatmap. Expression of glycolysis-associated genes increases along prognostic index, whereas that of fatty acid metabolism-associated genes decreases. OS, overall survival; HIF, hypoxia-inducible factor-1; FA, fatty acid.

Supplementary Table 1 Results of correlation analysis between expression of metabolism-associated genes and tumor FDG uptake in HCC.
