# Supplementary figures and images for: Metabolism-Associated Gene Signatures for FDG Avidity on PET/CT and Prognostic Validation in Hepatocellular Carcinoma
Source: Front Oncol. 2022 Jan 31;12:845900. doi: 10.3389/fonc.2022.845900 (PMC8841806; doi:10.3389/fonc.2022.845900)

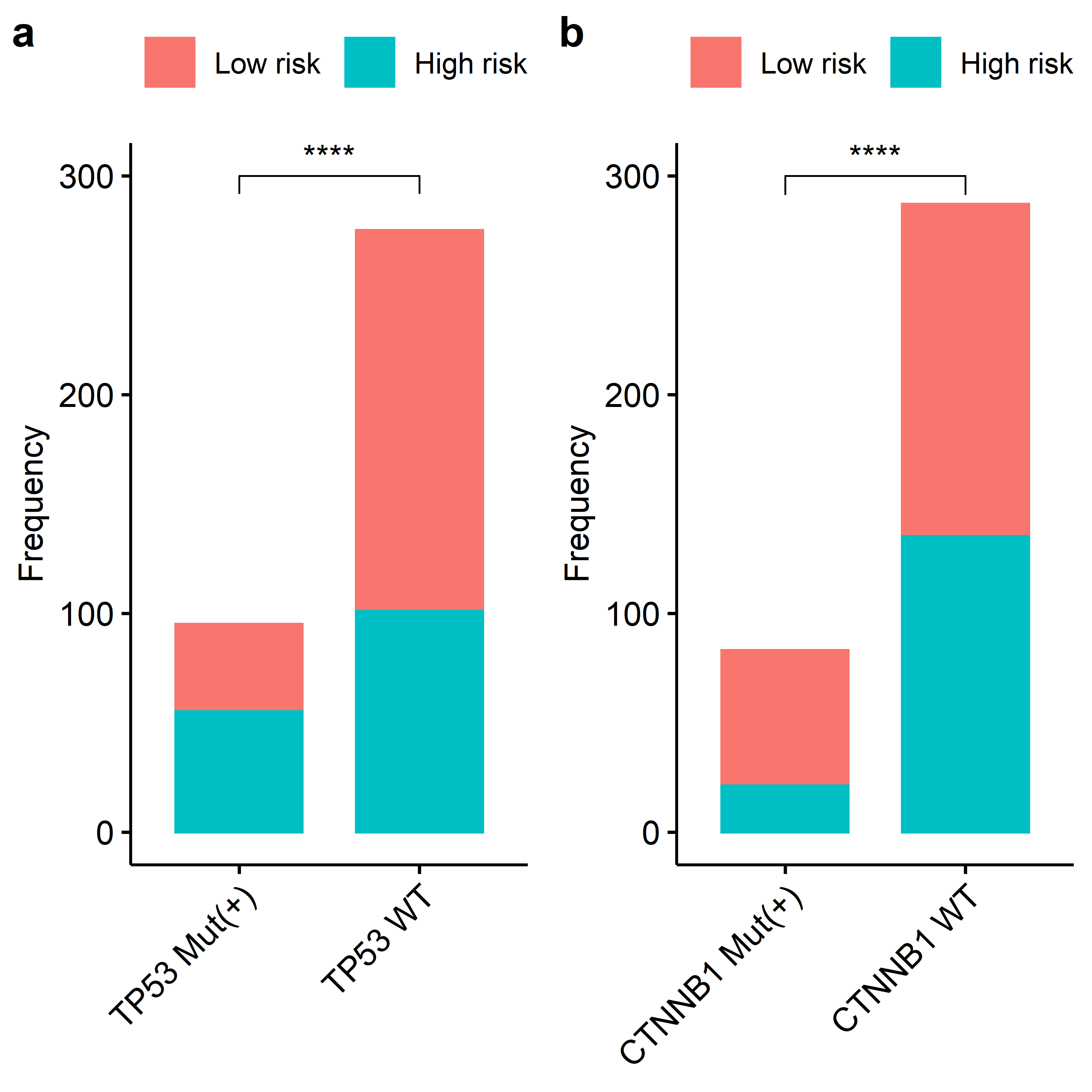

Supplement: Supplementary Figure 1 — The proportion of high-risk patients according to TP53 and CTNNB1 mutation status. (A) The proportion of high-risk patients with TP53-mutant HCC was significantly higher than that of those with wild-type TP53 (57.9% vs. 36.7%, p < 0.001). (B) The proportion of low-risk patients with CTNNB1-mutant HCC was significantly higher than that of patients with wild-type CTNNB-1 (74.7% vs. 53.0%, p < 0.001). Mut (+), mutant; WT, wild type. [file Image_1.tiff]

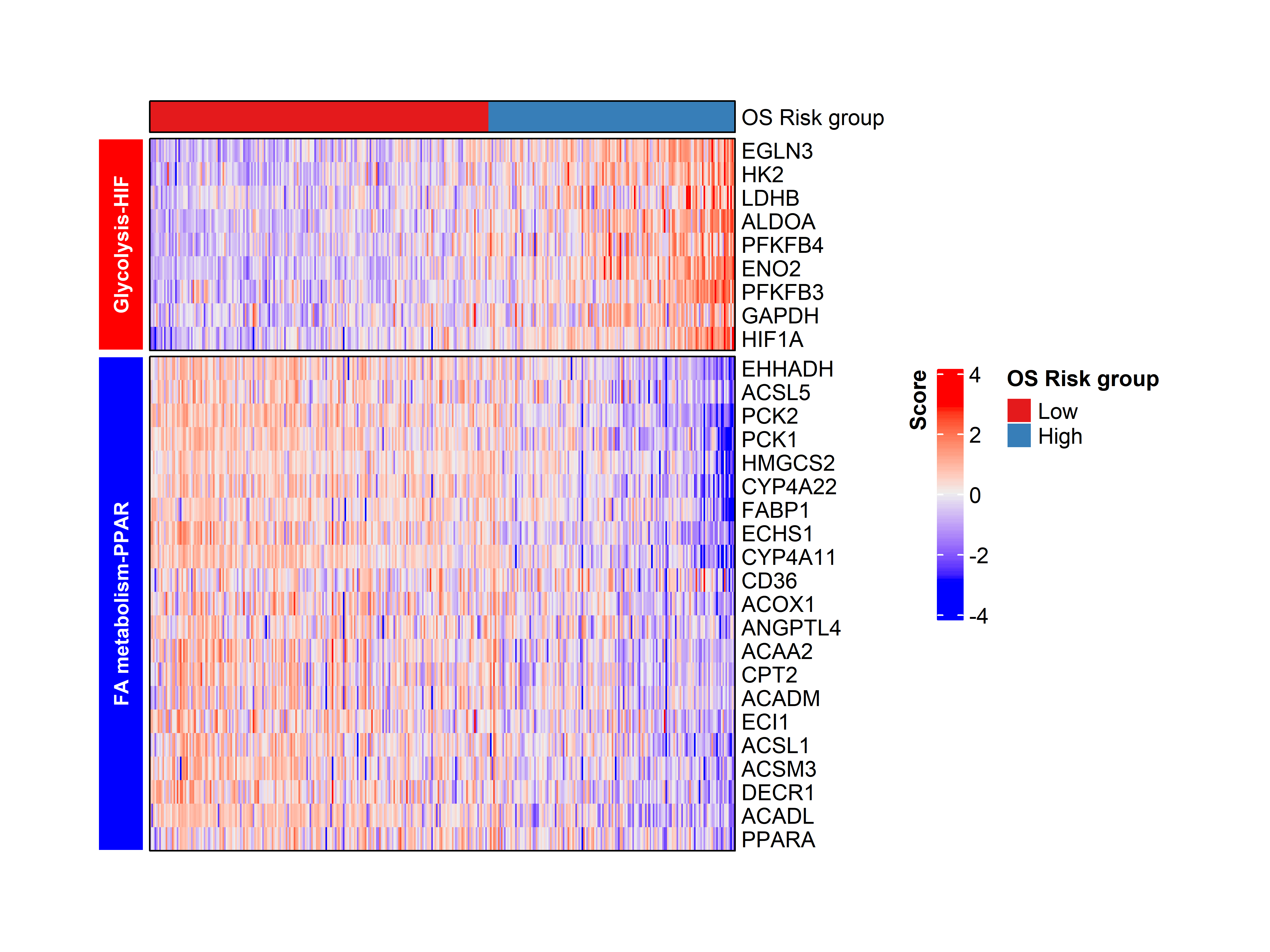

Supplement: Supplementary Figure 2 — The expression of 30 metabolism-associated genes according to prognostic index. Subjects were plotted in increasing order of prognostic index in the heatmap. Expression of glycolysis-associated genes increases along prognostic index, whereas that of fatty acid metabolism-associated genes decreases. OS, overall survival; HIF, hypoxia-inducible factor-1; FA, fatty acid. [file Image_2.tiff]
