# Supplementary material for: Metabolism-Associated Gene Signatures for FDG Avidity on PET/CT and Prognostic Validation in Hepatocellular Carcinoma
Source: Front Oncol. 2022 Jan 31;12:845900. doi: 10.3389/fonc.2022.845900 (PMC8841806; doi:10.3389/fonc.2022.845900)
Supplement: Supplementary file 4 [file DataSheet_1.docx]

**Supplementary Table 1. Results of correlation analysis between expression of metabolism-associated genes and tumor FDG uptake in HCC**

The excel file was attached.

**Supplementary Table 2. Metabolism-associated genes related to tumor FDG uptake involved in glycolysis and HIF-1 signaling.**

| **Gene** | **correlation coefficient** | ***p*-value** |
| --- | --- | --- |
| ALDOA | 0.376 | 0.003 |
| EGLN3 | 0.616 | < 0.001 |
| ENO2 | 0.342 | 0.007 |
| GAPDH | 0.299 | 0.020 |
| HK2 | 0.535 | < 0.001 |
| LDHB | 0.498 | < 0.001 |
| PFKFB3 | 0.315 | 0.014 |
| PFKFB4 | 0.349 | 0.006 |
| HIF1A | 0.258 | 0.047 |

**Supplementary Table 3. Metabolism-associated genes related to tumor FDG uptake involved in fatty acid metabolism and PPAR signaling**

| **Gene** | **correlation coefficient** | **P** |
| --- | --- | --- |
| ACAA2 | -0.351 | 0.006 |
| ACADL | -0.290 | 0.025 |
| ACADM | -0.332 | 0.010 |
| ACOX1 | -0.369 | 0.004 |
| ACSL1 | -0.316 | 0.014 |
| ACSL5 | -0.487 | < 0.001 |
| ACSM3 | -0.303 | 0.019 |
| ANGPTL4 | -0.354 | 0.005 |
| CD36 | -0.374 | 0.003 |
| CPT2 | -0.332 | 0.009 |
| CYP4A11 | -0.376 | 0.003 |
| CYP4A22 | -0.383 | 0.003 |
| DECR1 | -0.292 | 0.024 |
| ECHS1 | -0.377 | 0.003 |
| ECI1 | -0.328 | 0.011 |
| EHHADH | -0.491 | < 0.001 |
| FABP1 | -0.378 | 0.003 |
| HMGCS2 | -0.387 | 0.002 |
| PCK1 | -0.418 | < 0.001 |
| PCK2 | -0.448 | < 0.001 |
| PPARA | -0.265 | 0.041 |
